# Study of the temperature-related factors that activate the NLRP3 inflammasome in *Trichophyton schoenleinii* and their activation mechanisms

**DOI:** 10.3389/fcimb.2026.1823190

**Published:** 2026-05-20

**Authors:** Kaidiliya Aimilajiang, Xiaodong Wang, Mingshuo Ji, Palida Abulizi

**Affiliations:** Department of Dermatology, The First Affiliated Hospital of Xinjiang Medical University, Urumqi, Xinjiang, China

**Keywords:** dermatophyte, fungal pathogenicity, heat stress response, *T. schoenleinii*, transcriptome analysis

## Abstract

**Introduction:**

To investigate whether the temperature regulatory factor of *Trichophyton schoenleinii* (*T. schoenleinii*) is involved in the activation of the NLRP3 inflammasome and the regulation of related signaling pathways and to elucidate the unique pathogenesis of tinea capitis in Xinjiang.

**Methods:**

*T. schoenleinii* (strain 30076) isolated from patients with favus was subjected to heat stress at 42 °C and 37 °C, with normal temperature (27 °C) used as a control. Transcriptome sequencing and bioinformatics analysis were performed. A key temperature regulatory factor was screened, and a silencing strain was constructed. THP-1 cells were treated with a ROS inhibitor, a cathepsin inhibitor, high potassium, or RNA interference targeting NLRP3 and ASC and then infected with the wild-type or strains. The expression of IL-1β, IL-18, NLRP3, ASC, and Caspase-1 was detected.

**Results:**

Differentially expressed genes under heat stress were enriched in metabolic pathways and heat shock protein-related genes. TRINITY_DN3793_c0_g1 was identified as a temperature regulatory factor. *T. schoenleinii* induced the expression of IL-1β, IL-18, and NLRP3-related molecules in THP-1 cells, a process regulated by ROS, cathepsin, and potassium efflux. The temperature regulatory factor participated in but was not the sole requirement for NLRP3 inflammasome activation. The expression of related factors and ASC oligomerization were significantly lower in the silenced strain group than in the wild-type group.

**Discussion:**

Increased thermal stress causes severe mycelial damage in *T. schoenleinii*. Transcriptomics identifies TRINITY_DN3793_c0_g1 as a key temperature regulator. Infection activates the NLRP3 inflammasome via ROS, cathepsin B, and potassium efflux, promoting IL-1β/IL-18 secretion and ASC oligomerization. This regulator participates but is not essential for inflammasome activation. Findings reveal molecular links between temperature adaptation and pathogenicity, offering insights into tinea capitis pathogenesis and therapeutic targets.

## Graphical Abstract

**Figure:**
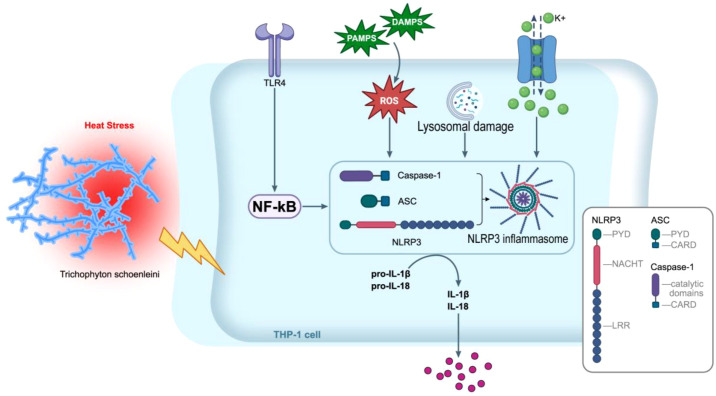
Regulatory factors and mechanism diagram of *Trichophyton schoenleinii*: temperature-dependent activation of the NLRP3 inflammasome

## Introduction

Dermatophytosis is a group of fungal infections caused by dermatophytes that infect the skin, hair, and nails. It primarily involves three genera, *Trichophyton, Microsporum*, and Epidermophyton, whose clinical manifestations include tinea capitis, tinea corporis, tinea cruris, tinea manuum and pedis, and tinea unguium (Deng et al., 2024). Epidemiological studies indicate that *Trichophyton rubrum* is the most common pathogen (accounting for 50~70%), followed by *Trichophyton mentagrophytes* (approximately 15~25%) (M M et al., 2021; Takashi et al., 2022). Dermatophyte infections exhibit distinct geographical distribution characteristics: the proportion of *T. rubrum* is higher and is continuously increasing in humid and hot regions, and the incidence rate is higher in the south than in the north and is higher in rural areas than in cities, possibly because of sanitary conditions and healthcare awareness. Tinea manuum and pedis and tinea unguium have high incidence rates and are increasing in prevalence among middle-aged and elderly people (Li et al., 2024). Tinea capitis affects mainly children, whereas tinea cruris is more common in males. Occupational groups such as athletes, soldiers, and miners have significantly higher incidences of tinea manuum and pedis because of hyperhidrosis, enclosed environments, and minor trauma. The persistent prevalence of dermatophyte infections is closely related to factors such as the weak capacity for fungal microscopy and culture at primary medical institutions, increased drug resistance due to irregular medication use, and insufficient public health education (Diba et al., 2024). *Trichophyton schoenleinii*, an anthropophilic dermatophyte, is the main pathogen of favus. Although it was once widespread and has now significantly decreased in most countries, it remains sporadic in certain regions (Z. M et al., 2024). Dry climates, limited sanitary conditions, and close contact transmission (e.g., sharing combs, hats, and towels) may be the main reasons for its high incidence in these areas (Fillipe et al., 2021).

Favus caused by *T. schoenleinii* is characterized by sulfur-yellow scutula, which can lead to permanent hair loss and atrophic scarring in severe cases. Its pathogenic mechanism involves the secretion of keratinase to decompose keratin for invasion into hair and epidermis and evasion of host immune clearance through long-term latency (Seetan et al., 2025). Some patients may experience significant inflammatory reactions, such as kerion. The diagnosis relies on fungal culture (slow growth on Sabouraud’s medium, waxy and folded colonies) and dermoscopy (typical sulfur-yellow scutula and atrophic scars). Treatment primarily involves systemic antifungal drugs (e.g., terbinafine) supplemented with topical antifungal agents and measures such as shaving hair and disinfecting utensils. Although *T. schoenleinii* infection is declining globally (Guan zhao et al., 2020), its public health impact in specific regions cannot be ignored. In China, *T. schoenleinii* is isolated mainly in the northwestern region, with sporadic findings in the south-central area. In recent years, cases have shown an increasing trend in some regions. However, its infection mechanism and virulence factors remain poorly understood.

The NLRP3 inflammasome plays a critical role in inflammatory responses and adaptive immunity. As a multiprotein complex, it recognizes pathogen-associated molecular patterns (PAMPs) or danger-associated molecular patterns (DAMPs) and activates Caspase-1, which subsequently cleaves pro-IL-1β and pro-IL-18 into their mature forms for release while also inducing pyroptosis (Zhu et al., 2025). Studies have shown that fungi such as *Cryptococcus neoformans* and *Candida albicans* can activate the NLRP3 inflammasome (Zheng et al., 2023). Preliminary research revealed that *T. schoenleinii* infection of THP-1 cells rapidly induces IL-1β secretion. Notably, heat-killed *T. schoenleinii* retains this ability, albeit at a reduced level compared with that of live fungi, suggesting the presence of heat-stable components within the fungus that participate in inflammasome activation (Liang et al., 2026). Therefore, it is hypothesized that temperature-sensitive components might be key factors in its virulence. The NLRP3 inflammasome can be activated by various danger signals, including infection and metabolic disorders (Chen et al., 2023). Its regulatory mechanisms are complex: autophagy negatively regulates its activity, whereas mitochondrial dysfunction leading to the accumulation of reactive oxygen species has a positive regulatory effect (Chen et al., 2024; Albright and Holian, 2025). Potassium ion efflux is a necessary upstream event for NLRP3 activation and is possibly related to conformational changes triggered by decreased intracellular potassium concentrations (Xu et al., 2024). Furthermore, NLRP3 is involved in Th17 cell differentiation and plays an important role in adaptive immune regulation (Leng et al., 2023). The fact that aluminum adjuvants can activate NLRP3 also suggests its potential value in vaccine development.

Fungal virulence is closely associated with thermotolerance. *Aspergillus fumigatus* can grow at 55 °C, and deficiencies in its thermotolerance-related genes (ribosome biogenesis protein CgrA, mannosyltransferase Pm1, and the functionally unknown protein Th1A) can affect its thermotolerance to varying degrees (Fabri et al., 2021). The ability of *C. neoformans* to grow at 37 °C is a prerequisite for its pathogenicity, with the Ras/PKA signaling pathway and the Rom2 protein playing key roles (Hu et al., 2021). In dermatophytes, the heat shock protein Hsp90 can mediate cell wall remodeling by regulating MAPK signaling, which plays a central role in the morphological transition and virulence formation of *C. albicans* (Chumakova et al., 2025). Previous studies have also shown that heat-treated *C. neoformans* capsule-deficient strains can still induce IL-1β secretion (Stempinski et al., 2021). These lines of evidence collectively suggest the universal importance of temperature regulators in fungal pathogenic mechanisms. On this basis, the present study proposes the following hypothesis: *T. schoenleinii* possesses temperature regulators that influence host immune responses by modulating NLRP3 inflammasome activation and related signaling pathways, thereby potentially explaining the inflammatory mechanisms of tinea capitis in certain regions.

To test this hypothesis, we used transcriptome sequencing to screen for genes that are differentially expressed under different temperatures, focusing on candidate factors related to virulence and the temperature response, such as heat shock proteins and metabolic enzymes. Gene silencing technology will be employed to construct target gene knockdown strains. In combination with dose-gradient treatments, interventions involving ROS/cathepsin inhibitors/potassium ions, and NLRP3/ASC gene-silenced cell lines, the role of these factors in Caspase-1 activation, IL-1β/IL-18 release, and ASC oligomerization will be systematically evaluated to elucidate their interaction mechanism with the NLRP3 inflammasome. From the perspective of pathogen–host interactions, this study aims to elucidate the molecular basis of the specific inflammatory phenotype of tinea capitis, not only increasing the understanding of *T. schoenleini* pathogenesis but also providing a theoretical foundation for therapeutic strategies targeting the NLRP3 pathway (such as the development of small molecule inhibitors) and immune interventions for tinea capitis. Preliminary findings that heat-killed *T. schoenleinii* can still activate NLRP3 suggest the direct involvement of heat-stable components in activation. On this basis, this project delves into the specific mechanism of action of temperature regulators, suggesting new directions for the precise prevention and treatment of tinea capitis.

## Materials and methods

### Fungal strains and culture conditions

In this study, *T. schoenleinii* strain 30076 was isolated from the hair of a patient with tinea favosa and preserved in the Mycology Lab of the Dermatology Department of the First Affiliated Hospital of Xinjiang Medical University. The strain was cultured on Sabouraud dextrose agar (Oxoid Ltd., Basingstoke, United Kingdom) at room temperature for 10 to 14 days. For heat shock treatment, the *T. schoenleinii* strains in the treatment group were sealed with parafilm and floated in a circulating thermoregulated water bath at 37 ± 0.05 °C for physiological temperature treatment and at 42 ± 0.05 °C for heat shock treatment for 30 min. Each treatment consisted of six replicates. After heat shock treatment, the fungi were reinoculated on fresh SDA plates to examine the effects of temperature on the growth or viability of *T. schoenleinii*, which were subsequently placed in incubators for the experiments (Experimental workflow, **Supplementary Figure 1**).

### RNA extraction, cDNA library construction and Illumina sequencing

After 30 min of heat exposure, total RNA was extracted via TRIzol reagent (Invitrogen, USA) following the manufacturer’s protocol, and DNase I (Takara, Japan) was used to remove genomic DNA. For whole RNA sequencing, approximately 4 µg (100 ng/µl) of high-quality total RNA (OD260/280 ≥ 2.0; RIN value ≥ 6.5) was extracted from each *T. schoenleinii* strain. The extracted RNA samples were sent to Beijing Allwegene Gene Technology Co. (Beijing, China) for further sequencing and analysis. After the sample is tested and qualified, the mRNA should be enriched via a specific method. For eukaryotes, magnetic beads with Oligo(dT) are used for enrichment. Following enrichment, the mRNA was fragmented into short pieces with fragmentation buffer. The first cDNA strand was synthesized via the use of random hexamer primers for reverse transcription, with cleaved RNA fragments serving as templates. Double-stranded cDNA was synthesized by adding buffer, dNTPs and DNA polymerase I. The double-stranded cDNAs were subsequently purified and eluted with elution buffer (EB) for end repair and poly(A) addition. Sequencing adapters were ligated onto the 5′ and 3′ ends of the fragments. AMPure XP beads were used to select the fragment size of the double-stranded cDNA. The fragments were purified via agarose gel electrophoresis and enriched via PCR amplification to create a cDNA library.

### Sequencing read quality and trimming

The quality of the sequencing data was assessed in terms of two aspects: sequence error rate distribution and base content distribution. Raw data usually contain a small amount of joint contamination and low-quality reads, which affects subsequent analysis if not filtered. High-quality, clean reads for the assembly library were generated by filtering according to the following rules: Filter reads with sequencing adapters. More than 10% of the unknown nucleotides (N) were filtered and reads containing more than 50% low-quality (Q ≤ 20) bases were filtered out.

### Transcriptome *de novo* assembly

For projects without reference genomes, the quality-filtered reads obtained were then *de novo* assembled into contigs via the Trinity Program (Grabherr et al., 2011) (https://github.com/trinityrnaseq/trinityrnaseq/wiki) for clean reads. Contigs and singletons were generated via *de novo* assembly, ultimately generating unigenes. This analysis provides the basis for subsequent gene expression and biological function analyses. After splicing with Trinity, the assembly results need to be optimized, filtered, and evaluated.

### Statistics of gene expression levels

The read count number of each sample for each gene was obtained, and FPKM conversion was performed. Afterward, the gene expression level was analyzed. In RNA-Seq technology, the expected number of fragments per kilobase of transcript sequence per million base pairs sequenced (FPKM) is the number of fragments per thousand base lengths from a gene per million fragments. The effects of sequencing depth and gene length on fragment count were also considered; this is the most commonly used method to estimate gene expression levels. Therefore, we performed FPKM conversion to read count numbers.

### Identification of DEGs

After the gene expression levels were determined, the expression data were analyzed statistically, and genes whose expression differed significantly between different samples were screened. The difference analysis was divided into three steps. First, the original read count was normalized, and the sequencing depth was corrected. The hypothesis testing probability (P value) is subsequently calculated via a statistical model. Finally, multiple hypothesis testing is performed.

Differential expression analysis was performed with the DESeq2 package19 (v1.28.1) between two different groups (*T. schoenleinii* cultured at room temperature as a control). The P values were subjected to false discovery rate (FDR) correction via the Benjamini–Hochberg method. Genes with an FDR ≤ 0.05 and a log2-fold change ratio ≥1 or ≤−1 were defined as differentially expressed genes (DEGs).

### Differential gene screening

A volcano map can be used to show the overall distribution of differentially expressed genes. The abscissa represents the change in gene expression log2(fold change) in different samples. The ordinate indicates the level of significance (-log10padj) of differences in expression. The upregulated genes are expressed in red; the downregulated genes are expressed in green.

### Functional annotation

By grouping genes that express the same or similar patterns into classes, to identify the functions of unknown genes or unknown functions of known genes, we used the BLASTx program (https://blast.ncbi.nlm.nih.-gov/Blast.cgi) with an E value threshold of 1×10^5^ to obtain protein functional annotations by aligning our unigenes to protein sequences from NCBI NR (nonredundant protein database, https://blast.ncbi.nlm.nih.gov/Blast.cgi), Swiss-Prot (annotated protein sequence database, http://www.expasy.org/), KEGG (Kyoto Encyclopedia of Genes and Genomes, http://www.genome.jp/kegg/), and COG (clusters of orthologous groups of proteins, https://www.ncbi.nlm.nih.gov/COG/). The Blast2GO program was used to obtain gene ontology (GO) annotations of our unigenes from the NR annotation, and then, GO seq, top GO software was used to perform GO functional classification. KEGG is a major public pathway-related database with which one can analyze gene products within the context of metabolic and cellular processes.

### GO and KEGG enrichment analyses of differentially expressed genes

GO (Gene Ontology, http://www.geneontology.org/) is an internationtology that includes Molecular Function/Biological Process/Cellular Component molecular function/biological process/cellular component. The basic unit of GO is the term, and each term corresponding to a function or attribute GO term with corrected P values of less than 0.05 was considered significantly enriched. KEGG (Kyoto Encyclopedia of Genes and Genomes) is a database for the systematic analysis of gene function and genomic information. As the primary public database related to pathways, the query of integrated metabolic pathways provided by KEGG, including carbohydrate, nucleoside, and amino acid metabolism and organic biodegradation, is very excellent; not only do they provide all possible metabolic pathways, but the enzymes that catalyze each reaction step are also comprehensively annotated. It is a powerful tool for metabolic analysis and metabolic network research *in vivo*.

*In vivo*, different genes coordinate with each other to perform their biological functions, and significant enrichment of pathways can reveal the main biochemical metabolic pathways and signal transduction pathways associated with the DEGs. Pathway enrichment analysis was conducted to identify pathways that were significantly enriched in DEGs compared with the entire transcriptome background by using a hypergeometric test with pathways in the KEGG database as units.

### Cell growth curve determination

The THP-1 cells in good growth conditions and at approximately 90% density were selected, and a single-cell suspension was prepared at a density of 3×10^4^ cells/mL in complete medium. One hundred microliters of the cell suspension was seeded into a 96-well plate every 24 hours (h). After 8 days of culture, 10 µL of preprepared CCK-8 solution was added to each well. The 96-well plate was incubated at 37 °C for 1 h, after which the optical density (OD) of each well was measured at a wavelength of 450 nm via a microplate reader. The cell growth curve was plotted on the basis of the OD values.

### Determination of the optimal MOI

The following groups were established: a blank control group, a *T. schoenleinii* wild-type strain intervention group, and a *T. schoenleinii* silenced strain intervention group. T.sch-wt represents the wild-type strain of the *Trichophyton schoenleinii* strain, and T.sch-mut represents the *Trichophyton schoenleinii* temperature-regulated target gene (TRINITY3793-c0-g1) silencing strain. The live cultures of each fungal strain were ground and washed three times with sterile PBS. The washed suspensions were counted using a counting chamber. Activated human THP-1-derived macrophages were infected with different multiplicity of infection (MOI) gradients (MOI = 1, 5, 10, and 20) for an infection duration of 48 h. Then, the levels of IL-1β and IL-18 in the cell culture supernatant were measured to determine a suitable MOI. Each MOI experiment was repeated three times.

### Determination of the optimal intervention time

Groups and Intervention: A blank control group, a *T. schoenleinii* wild-type strain intervention group, and a *T. schoenleini*i silenced strain intervention group were established. The live cultures of each fungal strain were ground and washed three times with sterile PBS. The washed suspensions were counted. Activated human THP-1-derived macrophages were infected at an MOI of 20 for different durations: 2 h, 6 h, 12 h, 24 h, and 48 h. The level of IL-1β in the cell supernatant and caspase-1 enzyme activity in the cells were subsequently detected. Each time point experiment was repeated three times.

### Comparison of different hyphal treatment groups

Activated human THP-1-derived macrophages were divided into the following groups: the normal control group (no intervention). Wild-type strain intervention group. Silenced Strain Intervention group. The fungal hyphae intervention groups were further subdivided into four subgroups. T. sch Intervention Group (MOI = 20), T.sch + ROS Inhibitor (10 mM NAC) Intervention Group, T.sch + Cathepsin B Inhibitor (24 µM CA-074 Me) Intervention Group, T.sch + Potassium Ion Intervention (50 mM KCl) Group. The intervention time was 48 h. After the intervention, both the cells and the cell culture supernatants were collected for subsequent detection of various indicators.

### Optimization of siRNA transfection conditions, screening of optimal siRNAs, and experimental validation of ASC and NLRP3 protein silencing results

Within a specific range, siRNA concentrations of 0.01 μM and 0.1 μM were selected as the siRNA concentration. After the cells were transfected with these two concentrations for 24 or 48 h, the results were observed via fluorescence microscopy. Activated THP-1 macrophages were transfected with siRNA-NC, siRNA-NLRP3, or siRNA-ASC under the optimal transfection conditions, and a blank control group was prepared for each group (with 3 replicates for each group). After the intervention, the cells were collected and washed with PBS once, after which the cells were collected again. Total protein was extracted, and the protein expression of NLRP3 and ASC was detected via WB.

### ELISA analysis

After the activated human THP-1-derived macrophages were subjected to the experimental grouping described above, the cell culture supernatant was collected. The levels of the inflammatory cytokines IL-1β and IL-18 were detected via ELISA.

### qRT–PCR analysis of IL-1β, IL-18, Caspase-1, NLRP3, and ASC gene expression

The THP-1 cells were collected, and a single-cell suspension was prepared at a density of 5×10^4^ cells/mL in culture medium. The suspensions were seeded into 6-well plates, and their differentiation into macrophages was induced by treatment with 50 ng/mL PMA for 48 h. The cells were subsequently treated according to the experimental groups described above, with three replicate wells per group. After treatment, the culture medium was discarded. One milliliter of TRIzol reagent was added to each well to lyse the cells, ensuring that the cell layer was evenly covered with the reagent. The plate was gently swirled to facilitate complete cell lysis, and then the lysate was transferred to 1.5 mL microcentrifuge tubes. Following total RNA extraction, the mRNA expression levels of the IL-1β, IL-18, Caspase-1, NLRP3, and ASC genes were detected via quantitative real-time PCR (qRT–PCR).

### Western blot analysis

Following the interventions according to the experimental groups described above, activated human THP-1-derived macrophages were collected and washed once with PBS. Total protein was then extracted. The protein expression levels of pro-IL-1β, NLRP3, pro-Caspase-1, and ASC were detected via Western blotting.

### Statistical analysis

Statistical analyses were performed with SPSS version 19.0 (IBM Corporation, Chicago, USA) software; all the experimental data are shown as the mean ± SD, and the results were analyzed by one-way analysis of variance (ANOVA). P values < 0.05 indicated statistical significance.

## Results

### Effects of heat stress on the growth and ultrastructure of *T. schoenleinii*

**Figure 1:**
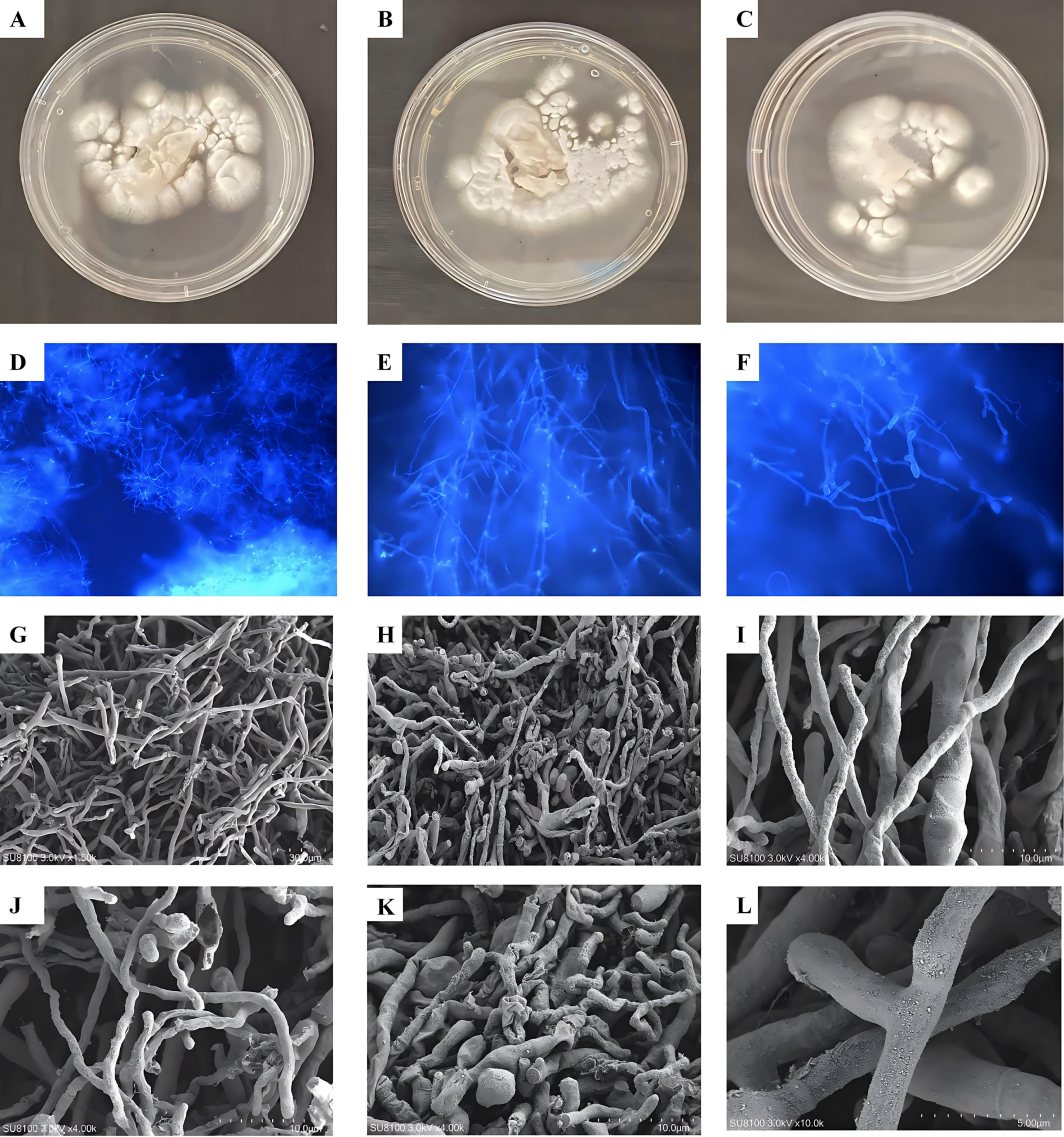
Colony growth and microscopic features of *T. schoenleinii* under different thermal conditions determined by scanning electron microscopy. **(A)** Colony growth of *T. schoenleinii* at room temperature, **(B)** Colony growth of *T. schoenleinii* at 37 °C. **(C)** Colony growth of *T. schoenleinii* under heat stress at 42 °C. **(D–F)** Microscopic morphology of *T. schoenleinii* at room temperature, 37 °C, and 42 °C, respectively. **(G–I)** Scanning electron microscopy images of *T. schoenleini*i at room temperature, 37 °C heat stress, and 42 °C heat stress, respectively (100× magnification). **(J–L)** Corresponding scanning electron microscopy images of *T. schoenleinii* at 200× magnification under the same thermal conditions.

In all three experimental groups, *T. schoenleinii* cells were initially cultured on SDA medium at 27 °C for 14 days. At room temperature (27 °C), *T. schoenleinii* grew well, forming folded, brain-like colonies that penetrated the culture medium (**Figure 1A**). Microscopic examination revealed typical features, including antler-like, swollen, and branched hyphae with abundant spores, while neither macroconidia nor microconidia were observed. Well-grown colonies were then subjected to heat treatment in a water bath at 37 °C and 42 °C for 30 min and subsequently recultured on SDA medium for two weeks. The colony morphology of the 37 °C-treated group was not significantly different from that of the room temperature (27 °C) group, although the growth rate was slower. In contrast, at 42 °C, the growth of *T. schoenleinii* was significantly inhibited compared with that in the room temperature group, as evidenced by a marked reduction in colony diameter (as shown in **Figures 1B, C**, respectively). Microscopic observation further demonstrated that both the 27 °C and 37 °C groups exhibited a typical antler-like hyphal morphology with swollen hyphae and abundant spores. At 42 °C, although the hyphal density was slightly reduced, the characteristic morphological structure remained identifiable (as shown in **Figures 1D–F**, respectively). These results indicate that elevated temperatures significantly inhibit the proliferation of *T. schoenleinii*, suggesting that temperature plays a crucial role in its growth.

To further investigate the effects of heat stress on the ultrastructure of *T. schoenleinii*, scanning electron microscopy was used to observe hyphal morphological changes under different treatment conditions. Under normal culture conditions (room temperature), the hyphae were robust, uniform, branched, and structurally intact, with smooth surfaces and no obvious damage, collapse, or shriveling (**Figures 1G, J**). Compared with the control group, *T. schoenleinii* subjected to heat stress at 37 °C exhibited curved growth and entangled hyphae. Damage to the hyphal surface was observed, including atrophy, uneven thickness, concavities, shrinkage, and visible cracks on the surface of some hyphae (**Figures 1H, K**). Under 42 °C heat stress, the morphological changes were even more pronounced: all the hyphae no longer maintained their original robust and uniform morphology. Instead, they displayed irregular and highly uneven structural features, with extreme entanglement among hyphae, wrinkled and rough surfaces, severe concavities in some hyphae, and twisting and folding of the mycelia themselves (**Figures 1I, L**). SEM observations further confirmed that heat stress not only inhibited the growth of *T. schoenleinii* but also severely damaged the ultrastructure of its hyphae, with the degree of damage intensifying as the temperature increased.

### Transcriptome sequencing analysis of the effects of temperature on gene expression in *T. schoenleinii*

**Figure 2:**
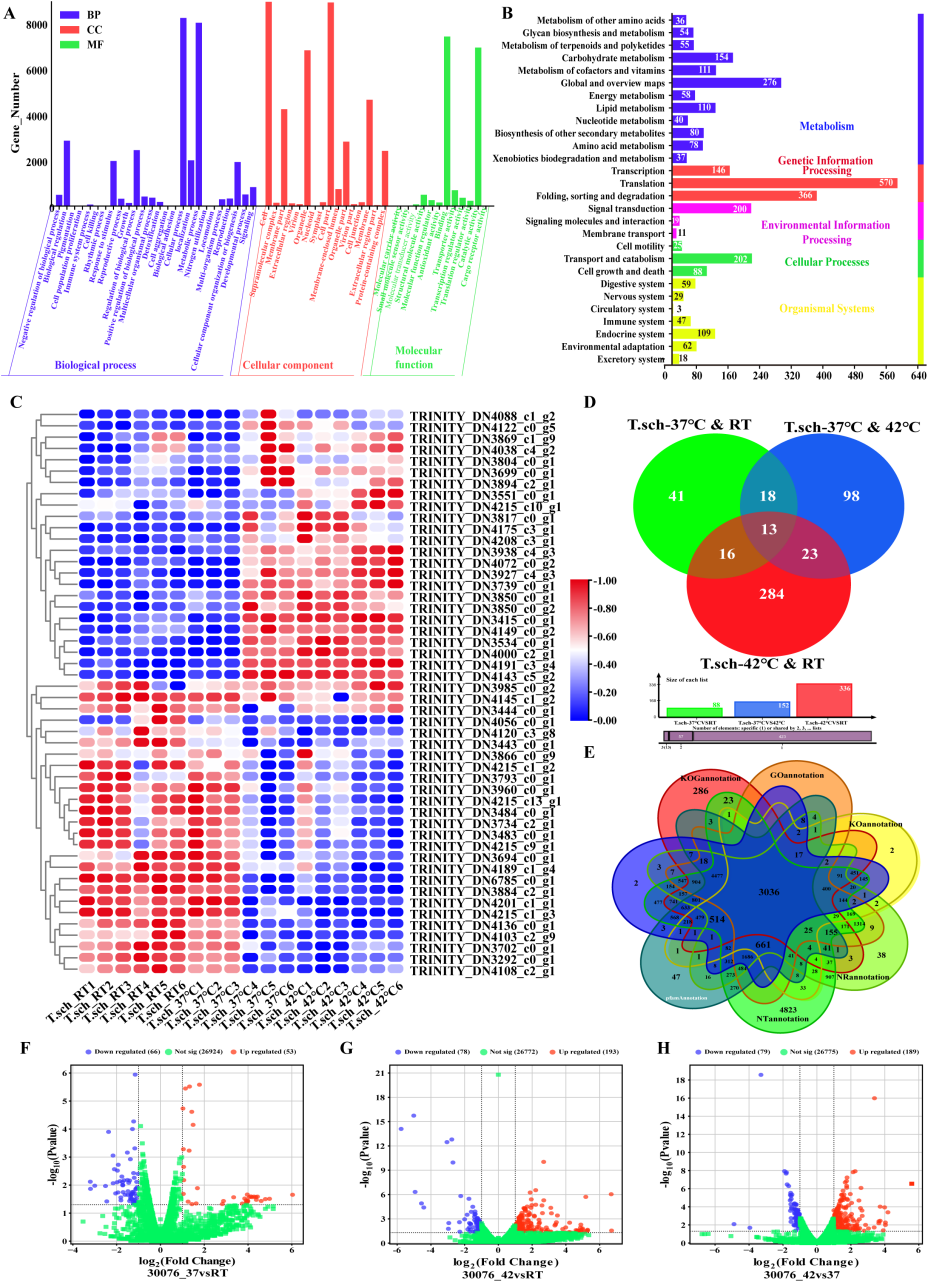
Transcriptomic analysis of *T. schoenleinii* under different temperatures. **(A)** GO functional classification of unigenes. **(B)** KEGG pathway classification of unigenes. **(C)** Cluster analysis of DEGs across the RT, 37 °C, and 42 °C groups. **(D)** Venn diagram showing common and unique DEGs among pairwise comparisons. **(E)** Venn diagram of unigene annotation statistics (NR, NT, Pfam, and GO). **(F–H)** Volcano plots of DEGs: **(F)** 37 °C vs RT, **(G)** 42 °C vs RT, **(H)** 42 °C vs 37 °C. Red: upregulated; blue: downregulated (padj < 0.05); green: not significant.

To investigate the impact of heat stress on gene expression in *T. schoenleinii*, transcriptome sequencing analysis was performed. All the clean reads were *de novo* assembled using Trinity software, generating 27043 unigenes. Through annotation against the NR, NT, KO, SwissProt, GO, and KOG databases, 17827, 26549, 6625, 17781, 14313, and 13227 unigenes were matched, respectively. The gene function annotation results revealed that 17827, 26549, 14916, and 14313 unigenes were enriched in the NR, NT, Pfam, and GO databases, respectively (**Figure 2A**).

GO functional enrichment analysis revealed that 14313 unigenes were classified into 54 functional categories, including 26 “biological process,” 12 “molecular function,” and 16 “cellular component” categories. Among the biological processes, “metabolic process” (8005 unigenes) and “cellular process” (8215 unigenes) was predominant, while “pigmentation” (1 unigene), “cell killing” (3 unigenes), and “cell aggregation” (1 unigene) were the least annotated. In the molecular function category, “binding” (7403 unigenes) and “catalytic activity” (6921 unigenes) accounted for the greatest proportions. With respect to cellular components, “cell” and “cell part” were the main categories, with “nucleoid” and “extracellular region part” being the least annotated. KEGG pathway enrichment analysis revealed that 3163 unigenes were annotated to 31 pathways (**Figure 2B**). Among these, the “metabolism” pathway category was the most common (1089 unigenes), primarily involving “global metabolism overview,” “carbohydrate metabolism,” “metabolism of cofactors and vitamins,” and “lipid metabolism.” This was followed by the “genetic information processing” category, which included “replication and repair,” “translation,” “folding, sorting and degradation,” and “transcription”. These pathways provide important resources for studying specific metabolic processes and gene functions in *T. schoenleinii.*

The cluster analysis of the DEGs revealed significant differences in gene expression among the various samples (**Figure. 2C**).

Following pairwise comparisons of differentially expressed genes (DEGs) common across experimental groups and subsequent combined analysis of shared DEGs, a total of 13 common DEGs were identified (**Figure. 2D**). As these genes present consistent expression changes in the same strain under temperature stress, it is hypothesized that these 13 genes may be associated with the heat stress response of strain 30076.

The basic functional annotation results of the genes revealed that a total of 17827 unigenes were enriched in the NR (NCBI nonredundant protein sequences) database, 26549 unigenes were enriched in the NT (NCBI nucleotide sequences) database, 14916 unigenes were enriched in the Pfam (protein family) database, and 14313 unigenes were enriched in the GO (Gene Ontology) database (**Figure. 2E**).

Differential expression gene (DEG) screening revealed that,compared with those in the room temperature group, 53 significantly upregulated and 66 significantly downregulated DEGs were detected in the 37 °C treatment group (**Figure 2F**); in the 42 °C treatment group,193 genes were significantly upregulated, and 78 were significantly downregulated (**Figure 2G**), When the 42°C and 37°C groups were compared directly, 189 upregulated and 79 downregulated genes were identified (**Figure. 2H**). Volcano plot visualization showing the distribution characteristics of the DEGs: the horizontal axis represents the log_2_ fold change in gene expression, and the vertical axis represents the -log_10_ (padj) value for significance. The upregulated genes are marked in red; the downregulated genes are marked in blue, and the genes whose expression is not significantly differentially expressed are marked in green. Hierarchical clustering analysis of differentially expressed genes revealed significant transcriptomic differences among samples under different treatment conditions, indicating that temperature treatment had a global effect on gene expression. On the basis of this expression profile pattern, a core set of 50 DEGs, namely, TRINITY_DN3793_c0_g1, TRINITY_DN3960_c0_g1, and TRINITY_DN4215_c13_g1, was identified through intergroup comparisons. These genes exhibited significant changes in expression across the different temperature treatments, suggesting that they may play key roles in the response of organisms to temperature stress. Further Venn diagram analysis of DEGs coexpressed in pairwise comparisons between experimental groups, followed by combined comparisons, revealed 13 commonly coexpressed DEGs. Given that these genes are commonly differentially expressed in the same strain under temperature stress, these 13 genes may be closely associated with the heat stress response of *T. schoenleinii.*

### Molecular mechanisms of *T. schoenleinii* in response to heat stress: functional enrichment analysis of differentially expressed genes

**Figure 3:**
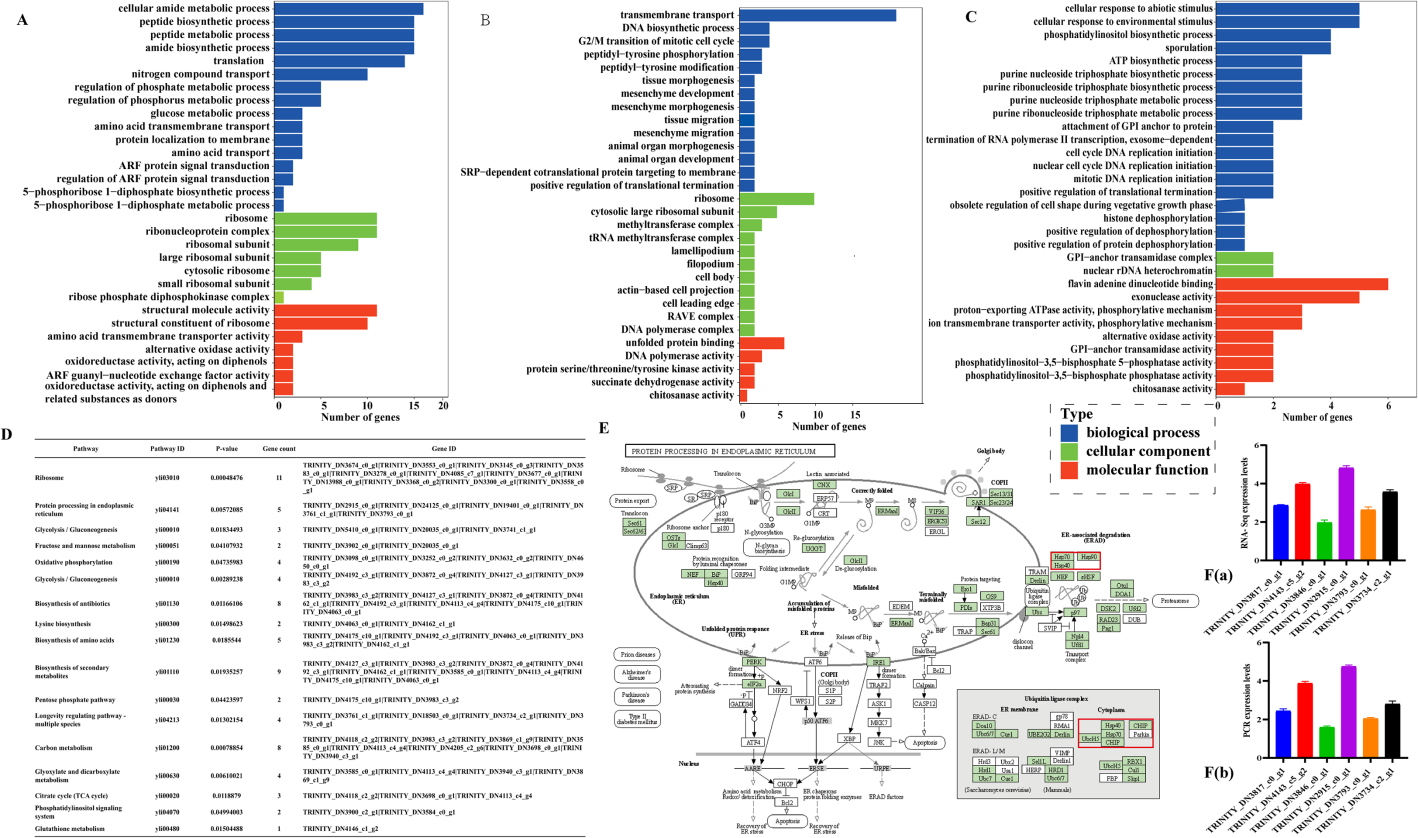
Functional enrichment analysis of differentially expressed genes and validation of the RNA-seq data by qRT–PCR. **(A)** GO enrichment analysis of DEGs between the 37 °C and room temperature groups. **(B)** GO enrichment analysis of DEGs between the 42 °C and 37 °C groups. **(C)** GO enrichment analysis of DEGs between the 42 °C and room temperature groups. **(D)** KEGG pathway enrichment analysis of DEGs, showing the most significantly enriched pathways. **(E)** Representative enriched pathways (protein processing in the endoplasmic reticulum) and interaction network of the HSP-related proteins. **(F)** Validation of RNA-seq data by qRT–PCR for selected DEGs, showing consistent expression trends between the two methods.

To investigate the molecular mechanisms underlying the heat stress response in *T. schoenleinii*, GO and KEGG enrichment analyses were performed on the differentially expressed genes. GO enrichment analysis revealed (as shown in **Figures 3A–C**, respectively) that in the 37 °C vs RT group, the DEGs were significantly enriched in terms related to translation, peptide metabolism, and ribosomal structural constituents. In the 42 °C vs 37 °C group, the significantly enriched terms included cellular response to stimuli, phosphatidylinositol biosynthesis, and flavin adenine dinucleotide binding. The 42 °C vs RT group was significantly enriched in functions such as transmembrane transport, DNA synthesis, unfolded protein binding, and DNA polymerase activity. KEGG enrichment analysis revealed 17 significantly enriched pathways (*P* < 0.05), including ribosome, protein processing in the endoplasmic reticulum, glycolysis/gluconeogenesis, oxidative phosphorylation, and longevity-regulating pathways, in multiple species (**Figure 3D**). With respect to protein processing in the endoplasmic reticulum pathway, multiple heat shock protein family members, such as Hsp40 (TRINITY_DN19401_c0_g1), Hsp90 (TRINITY_DN4143_c5_g2, among others), and other Hsp-related genes, were identified (**Figure 3E**). These molecular chaperones play crucial roles in assisting proper protein folding and maintaining cellular homeostasis.

On the basis of gene expression levels and pathway enrichment significance, six candidate heat stress-responsive genes were preliminarily screened: TRINITY_DN3817_c0_g1, TRINITY_DN4143_c5_g2, TRINITY_DN3846_c0_g1, TRINITY_DN2915_c0_g1, TRINITY_DN3793_c0_g1, and TRINITY_DN3734_c2_g1. qRT–PCR validation was subsequently performed on the six most significantly differentially expressed genes, and the results revealed that their expression trends were largely consistent with the transcriptome sequencing data, confirming the reliability of the results of the RNA–seq analysis (**Figure 3F**). Under 42 °C heat stress, the expression of this gene was significantly upregulated, ranking among the top three genes in the volcano plot, and its expression level increased progressively with increasing temperature (*P* < 0.001). The clustering heatmap revealed distinct expression patterns between groups and good reproducibility within groups. Additionally, this gene was enriched in key pathways such as endoplasmic reticulum protein processing, longevity regulation, endocytosis, and ribosome. In summary, TRINITY_DN3793_c0_g1 was selected as the core temperature-regulatory factor (**Supplementary Figure 2**).

### Mechanism of the regulation of the inflammatory response of THP-1 cells by temperature via the NLRP3 inflammasome

**Figure 4:**
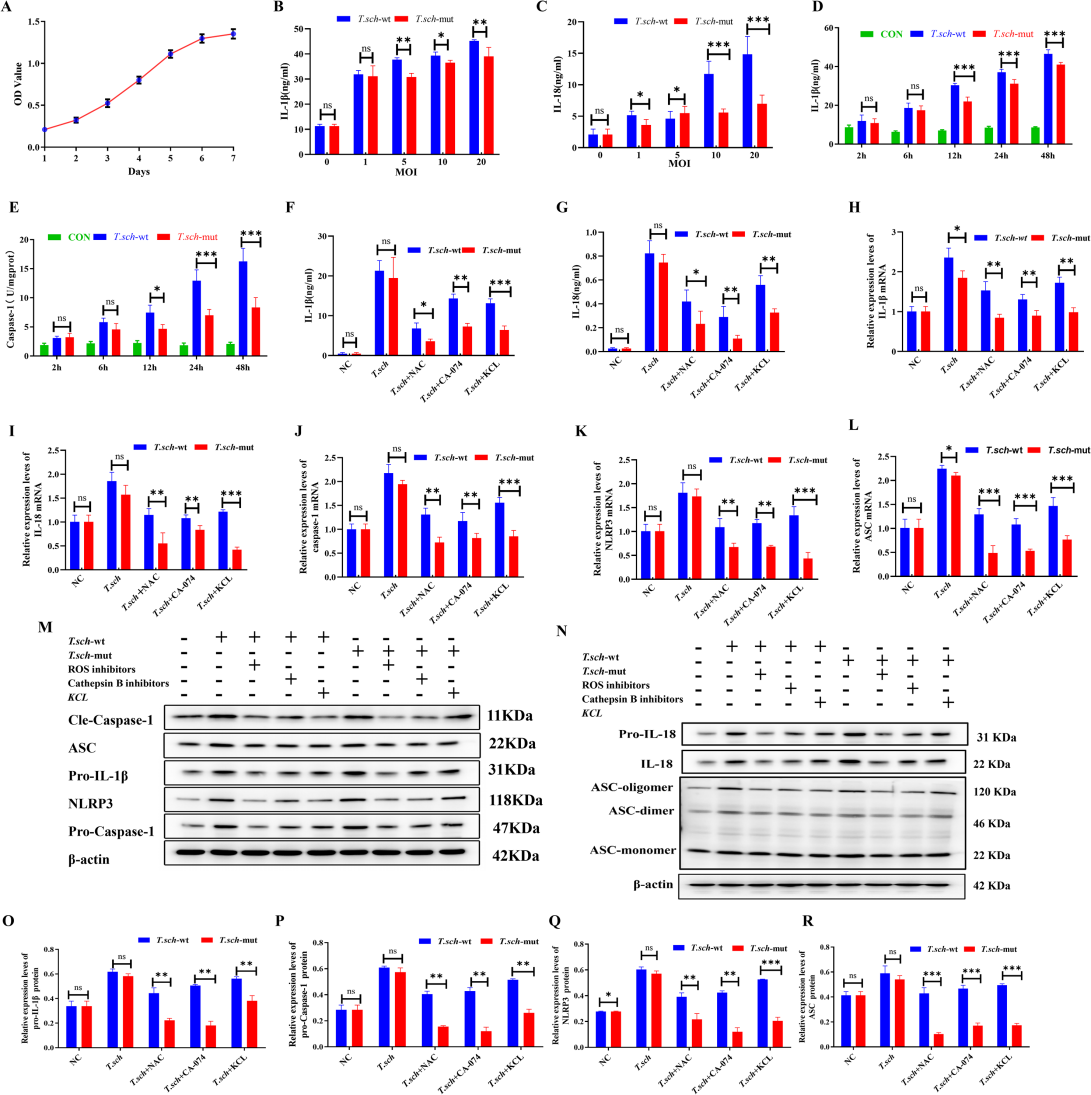
Role of target genes in NLRP3 inflammasome activation via the ROS, cathepsin B, and potassium efflux pathways. **(A)** THP-1 cell growth curve rate. **(B)** IL-1β secretion levels in THP-1 cell supernatants at different MOIs (0, 1, 5, 10, and 20) detected by ELISA. **(C)** IL-18 secretion levels in THP-1 cell supernatants at different MOIs (0, 1, 5, 10, and 20) detected by ELISA. **(D, E)** Kinetics of inflammasome activation. IL-1β levels in the supernatants **(D)** and intracellular caspase-1 activity **(E)** at the indicated time points (2–48 h) after infection with the wild-type or silenced strains (MOI = 20). **(F–G)** Effects of pathway inhibitors on cytokine secretion. IL-1β **(F)** and IL-18 **(G)** levels in the supernatants of THP-1 cells pretreated with a ROS inhibitor (NAC), a cathepsin B inhibitor (CA-074), or high-K^+^, followed by stimulation with the wild-type or silenced strains. **(H–K)** Effects of inhibitors on gene expression. qRT-PCR analysis of IL-1β **(H)**, IL-18 **(I)**, NLRP3 **(J)**, and ASC **(K)** mRNA levels in THP-1 cells under different treatment conditions. **(L–R)** Effects of inhibitors on protein expression. Western blot analysis of pro-IL-1β, NLRP3, pro-Caspase-1, mature Caspase-1, and ASC (monomer, dimer, and oligomer) expression in THP-1 cells under the indicated conditions. (^*^P < 0.05, ^**^P < 0.01, ^***^P < 0.001, ns = not significant).

To determine the optimal experimental conditions, the release levels of inflammatory factors from THP-1 cells were first assessed under different multiplicities of infection (MOIs) and infection times (**Figure 4A**). The results revealed that at an MOI of 20, the secretion of both IL-1β and IL-18 peaked, with that of IL-1β at 45.245 ± 0.404 pg/mL and that of IL-18 at 14.863 ± 2.816 pg/mL (**Figures 4B, C**). Which were significantly greater than those in the groups with MOIs of 0, 1, 5, and 10 (*P* < 0.05), indicating that an MOI of 20 was the optimal infection concentration. Cells were then infected at an MOI of 20, and IL-1β levels and caspase-1 activity were measured at different time points (2, 6, 12, 24, and 48 h). As shown in **Figures 4D, E**, at 48 h post infection, both the IL-1β level and the caspase-1 activity were significantly greater than those at the other time points (*P* < 0.05). Moreover, the IL-1β level (46.563 ± 2.100 pg/mL) and caspase-1 activity (16.263 ± 2.239) induced by the wild-type strain were significantly greater than those induced by the silencing strain (41.084 ± 1.096 pg/mL; 8.363 ± 1.671) demonstrating that the target gene is involved in the secretion of inflammatory factors in a time-dependent manner.

To investigate the specific mechanism by which the target gene activates the NLRP3 inflammasome, THP-1 cells were treated with an ROS inhibitor, a cathepsin B inhibitor, or high concentrations of KCl and then stimulated with hyphae from the wild-type or silenced strains. IL-1β and IL-18 levels were measured. The ELISA results revealed that under all three intervention conditions, the levels of IL-1β and IL-18 induced by the wild-type strain were significantly greater than those induced by the silenced strain (*P* < 0.05; **Figures 4F, G**, respectively), but both were lower than those in the untreated control group. qRT-PCR analysis further confirmed that compared with those in the wild-type strain group, the mRNA expression levels of IL-1β, IL-18, NLRP3, caspase-1, and ASC were significantly lower and that the expression levels in the silenced strain group were significantly lower (*P* < 0.05, as shown in **Figures 4H–L**, respectively). These results indicate that ROS production, cathepsin B release, and K^+^ efflux are important pathways through which *T. schoenleinii* activates the NLRP3 inflammasome and that the target gene participates in this process, although it is not the sole determining factor.

Western blot analysis was performed to detect changes in NLRP3 inflammasome-related protein expression. As shown in **Figures 4O–R**, compared with those in the blank control group, the expression of pro-IL-1β, pro-Caspase-1, NLRP3, and ASC was significantly greater in the group of cells stimulated with either the wild-type or silencing strain (*P* < 0.05), the expression of mature Caspase-1 was greater, and the formation of ASC oligomers (ASC oligomers and ASC dimers) was greater (**Figure 4N**). Furthermore, the levels of all these indicators were significantly greater in the wild-type strain group than in the silenced strain group. Additionally, treatment with ROS inhibitors, cathepsin B inhibitors, and high potassium concentrations significantly reduced the expression levels of mature caspase-1, NLRP3, and pro-IL-1β (*P* < 0.05), suggesting that these pathways inhibit NLRP3 inflammasome activation by suppressing pro-caspase-1 activation. In conclusion, *T. schoenleinii* infection induces pro-IL-1β expression, ASC oligomerization, and Caspase-1 activation in THP-1 cells (**Figure 4M**). The target gene is involved in this process but is not essential for NLRP3 inflammasome activation.

### Identification of core targets in the NLRP3-ASC interaction network and their regulatory roles in inflammatory pathways

**Figure 5:**
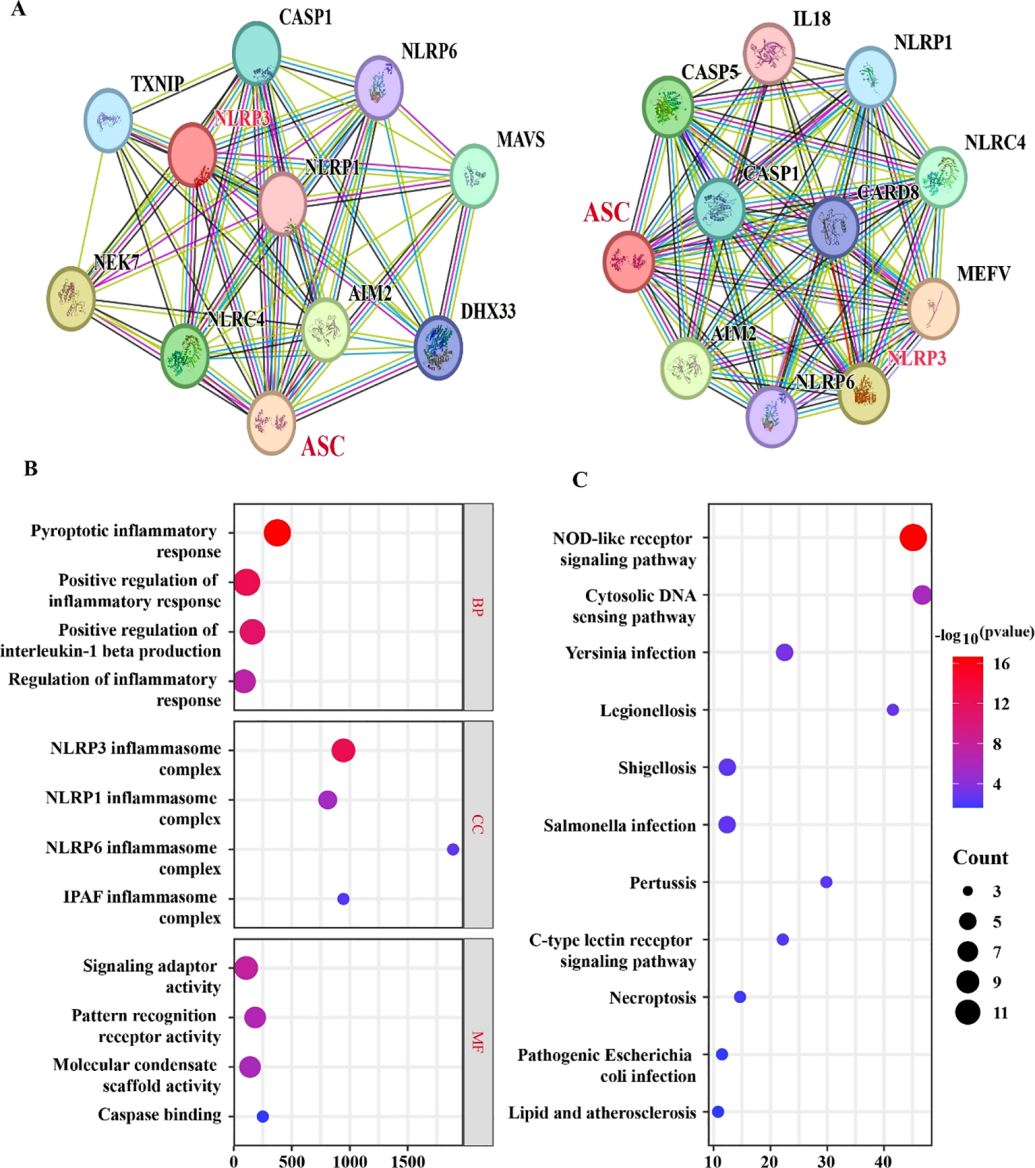
Bioinformatics analysis of the NLRP3-ASC interaction network and functional enrichment of differentially expressed genes. **(A)** Protein–protein interaction networks. Left: NLRP3 interaction network; Right: ASC interaction network. Nodes represent proteins, and edges represent predicted functional associations. **(B)** GO functional enrichment analysis of DEGs. **(C)** KEGG pathway enrichment analysis of DEGs.

To investigate the interactions between NLRP3 and ASC and their regulatory mechanisms in the heat stress response of *T. schoenleinii* in depth, a protein–protein interaction network was constructed using the STRING database. The analysis revealed that targets such as Casp1, ASC, NLRP3, NLRP1, IL-18, and Casp5 exhibited high degrees of connectivity, suggesting their central positions and key biological functions within this network (**Figure 5A**). Further GO functional enrichment analysis of the differentially expressed genes revealed that these genes were significantly enriched in biological processes related to the inflammatory response, immune response, and cell death pathways, including the pyroptotic inflammatory response, positive regulation of the inflammatory response, positive regulation of interleukin-1 beta production, and regulation of the inflammatory response (**Figure 5B**). In terms of cellular components, they were significantly enriched in the NLRP3, NLRP1, NLRP6, and IPAF inflammasome complexes. In terms of molecular functions, these genes were primarily involved in signaling adaptor activity, pattern recognition receptor activity, molecular condensate scaffold activity, and caspase binding. KEGG pathway enrichment analysis further demonstrated that the DEGs were significantly enriched in important pathways, such as the NOD-like receptor signaling pathway, cytosolic DNA-sensing pathway, Yersinia infection, Legionellosis, Shigellosis, Salmonella infection, Pertussis, C-type lectin receptor signaling pathway, Necroptosis, Pathogenic *Escherichia coli* infection, and Lipid and atherosclerosis (**Figure 5C**). These results systematically reveal the core nodes of the NLRP3-ASC interaction network and the inflammation-related biological processes and signaling pathways in which these genes participate, providing an important bioinformatics foundation for understanding the mechanism through which temperature-regulated genes in *T. schoenleinii* affect NLRP3 inflammasome activation.

### Effects of NLRP3/ASC gene silencing on *T. schoenleinii*-induced inflammasome activation in THP-1 cells

**Figure 6:**
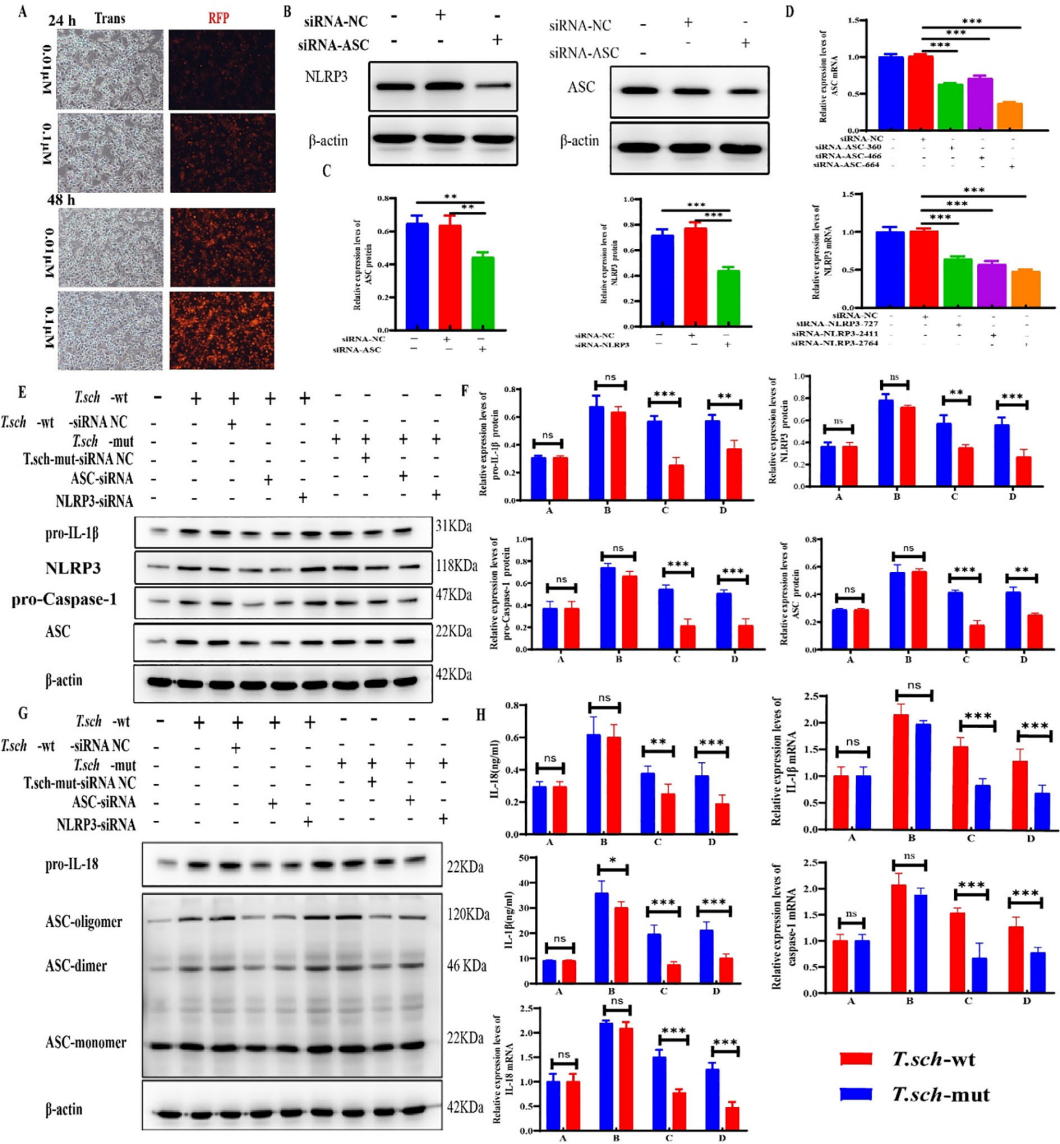
Verification of the role of temperature-regulated genes in NLRP3 inflammasome activation via RNA interference. **(A)** Fluorescence microscopy results of THP-1 cells transfected with 0.01 μM and 0.1 μM siRNA for 24 h and 48 h, respectively. **(B)** Western blot analysis of ASC and NLRP3 protein expression in THP-1 cells after transfection with ASC siRNA or NLRP3 siRNA. **(C)** Bar chart of the protein expression levels of ASC and NLRP3 in THP-1 cells after transfection with ASC siRNA or NLRP3 siRNA. **(D)** qRT–PCR analysis of ASC and NLRP3 mRNA expression in THP-1 cells after transfection with ASC siRNA or NLRP3 siRNA. **(E)** Western blot analysis of pro-IL-1β, pro-Caspase-1, NLRP3, and ASC protein expression in THP-1 cells under different treatment conditions. **(F)** Bar chart of the protein expression levels of pro-IL-1β, pro-Caspase-1, NLRP3, and ASC in THP-1 cells under different treatment conditions. **(G)** Western blot analysis of ASC oligomerization (ASC-oligomer, ASC-dimer, and ASC-monomer) in THP-1 cells under different treatment conditions. **(H)** qRT–PCR analysis of IL-1β, IL-18, Caspase-1, NLRP3, and ASC mRNA expression levels in THP-1 cells under different treatment conditions. (^*^P < 0.05, ^**^P < 0.01, ^***^P < 0.001, ns = not significant).

To determine the optimal transfection conditions, THP-1 cells were transfected with 0.01 μM and 0.1 μM siRNA for 24 h and 48 h, respectively. The results of fluorescence microscopy revealed that transfection with 0.1 μM siRNA for 48 h resulted in greater than 90% fluorescence intensity. Therefore, 0.1 μM siRNA and 48 h of transfection were selected as the optimal conditions for subsequent experiments (**Figure 6A**). Under these conditions, THP-1 cells were transfected with ASC siRNA or NLRP3 siRNA, and the silencing efficiency was validated by Western blotting and qRT-PCR. As shown in **Figures 6B, C**, compared with those in the negative control group, both the protein expression levels and the mRNA expression levels (**Figure 6D**) of ASC and NLRP3 in THP-1 cells significantly decreased after transfection with ASC siRNA and NLRP3 siRNA (*P* < 0.05), indicating the successful establishment of ASC and NLRP3 gene-silenced cell models. Changes in NLRP3 inflammasome-related indicators were further examined in THP-1 cells with successful ASC and NLRP3 gene silencing. Compared with those in the negative control group, the formation levels of ASC oligomers (ASC oligomers, ASC dimers, and ASC monomers) in the siRNA-NLRP3- and siRNA-ASC-transfected groups were significantly lower (*P* < 0.05). Moreover, the level of ASC oligomerization in the *T. schoenleinii*-silenced strain stimulation group was significantly lower than that in the wild-type strain stimulation group (*P* < 0.05; **Figure 6G**). Moreover, the protein expression levels of pro-IL-1β and pro-Caspase-1 were significantly lower in the gene-silenced groups (*P* < 0.05), with lower levels observed in the silencing strain group than in the wild-type strain group (**Figures 6E, F**). qRT–PCR analysis further confirmed that in THP-1 cells with NLRP3 or ASC gene silencing, the mRNA expression levels of IL-1β, IL-18, NLRP3, ASC, and Caspase-1 induced by *T. schoenleinii* were significantly lower (*P* < 0.05), and the expression levels of all the indicators in the silenced strain group were significantly lower than those in the wild-type strain group (**Figure 6H**). These results indicate that ASC oligomerization, the activation of pro-IL-1β and pro-Caspase-1, and the production of related inflammatory factors induced by *T. schoenleinii* in THP-1 cells are dependent on NLRP3 inflammasome assembly. The temperature-regulated gene may be involved in this activation process, but it is not the only necessary condition for NLRP3 inflammasome activation.

## Discussion

*Trichophyton schoenleinii* is an anthropophilic dermatophyte responsible for chronic infections such as tinea capitis (favus), particularly in endemic regions. Its pathogenicity involves multiple virulence factors, including keratinases, lipases, and other hydrolases that disrupt the skin barrier, facilitate tissue invasion, and promote nutrient acquisition (Celestrino et al., 2021; Peng et al., 2024; Miao et al., 2026). Additionally, immune evasion strategies such as cell wall masking and biofilm formation contribute to fungal persistence in host tissues. Although improved sanitation has shifted the global epidemiology of tinea capitis toward zoophilic species such as *Microsporum canis*, *T. schoenleinii* remains endemic in certain regions, including southern Xinjiang, China, highlighting the need to understand its pathogenic mechanisms (Tettey et al., 2024). Our team has long focused on this regional infection. Preliminary work revealed that heat-inactivated *T. schoenleinii* (90 °C, 30 min) still induced cytokine production in THP-1 cells, suggesting the presence of heat-stable components capable of activating the inflammasome.

In this study, *T. schoenleinii* strains were exposed to heat stress at 37 °C and 42 °C for 30 min. Heat stress significantly affected fungal growth and morphology, with colony growth inhibited in a temperature-dependent manner and more severe restriction at 42 °C. Scanning electron microscopy revealed increasingly irregular hyphal morphology, including surface wrinkling, depressions, and twisted, folded structures, particularly at higher temperatures. These findings suggest that temperature is an important environmental factor influencing fungal physiology and, potentially, its pathogenic capacity.

Transcriptome sequencing of heat-treated hyphae revealed substantial changes in gene expression between the temperature groups. Enriched pathways included protein processing in the endoplasmic reticulum, glycolysis/gluconeogenesis, fructose and mannose metabolism, oxidative phosphorylation, lysine biosynthesis, and longevity regulation. Among these genes, the most significantly upregulated genes encoded the heat shock proteins Hsp90, Hsp70, and Hsp40. Hsps are evolutionarily conserved molecular chaperones that are induced under stress conditions and play essential roles in maintaining protein homeostasis (Wu et al., 2023; Yingying et al., 2024). Their upregulation suggests an adaptive response that may increase fungal survival under host-related temperatures, but their role in *T. schoenleinii* has been poorly characterized. Compared with the room temperature control group, the 37 °C group had 53 upregulated genes and 66 downregulated genes, and the 42 °C group had 193 upregulated genes and 78 downregulated genes. Comparisons between 42 °C and 37 °C revealed 189 upregulated and 79 downregulated genes. After the results of the GO and KEGG analyses were performed, six candidate heat stress-responsive genes were validated by qRT-PCR, which confirmed the transcriptomic trends. One gene, TRINITY_DN3793_c0_g1, was identified as a key temperature-regulated target. A critical unresolved question is whether this gene influences host immunity by modulating NLRP3 inflammasome activation and related pathways, such as NF-κB or MAPK, potentially contributing to the unique inflammatory phenotype of tinea capitis observed in children from the Xinjiang region.

Previous research has confirmed that *T. schoenleinii*-induced IL-1β secretion depends on NLRP3 inflammasome activation (Chaudhary and Mishra, 2024; Owona et al., 2024). In our study, siRNA-mediated silencing of NLRP3 or ASC expression significantly reduced IL-1β secretion, ASC oligomerization, and mature caspase-1 expression, confirming that IL-1β release requires NLRP3 inflammasome activation. Using a THP-1-derived macrophage model, we observed that *T. schoenleinii* stimulation was associated with increased secretion of IL-1β and IL-18 and elevated expression of NLRP3, ASC, and caspase-1, suggesting that *T. schoenleinii* can induce inflammasome-related responses *in vitro*.

We also investigated the upstream mechanisms involved. Inhibiting cathepsin B, blocking K^+^ efflux with high concentrations of KCl, or suppressing ROS generation reduced the expression of mature IL-1β and caspase-1. ROS inhibition with NAC did not significantly reduce IL-1β secretion but did lower mature caspase-1 levels, reflecting the complex regulatory role of ROS, which may act through direct NLRP3 modification, mitochondrial function, or ion channel activity. Together, these results indicate that K^+^ efflux, lysosomal rupture, and ROS generation are involved in *T. schoenleinii*-mediated NLRP3 inflammasome activation.

To determine the role of this temperature-regulated gene, we constructed THP-1 cells treated with extracellular potassium, a ROS inhibitor, or a cathepsin B inhibitor and then stimulated them with wild-type or gene-silenced *T. schoenleinii* hyphae. Compared with the wild-type strain, the gene-silenced strain induced significantly lower IL-1β and IL-18 secretion and reduced NLRP3 and ASC mRNA expression (*P* < 0.05). In additional experiments, THP-1 cells were transfected with siRNA-NLRP3 or siRNA-ASC under optimal conditions (0.1 μM siRNA, 48 h). Silencing of either gene reduced IL-1β and IL-18 secretion, which is consistent with the results of the ELISA and qRT–PCR. Moreover, the mRNA levels of IL-1β, IL-18, caspase-1, NLRP3, and ASC were significantly lower in cells stimulated with the gene-silenced strain than in those stimulated with the wild-type strain (*P* < 0.05). Western blotting revealed decreased ASC oligomer formation in siRNA-NLRP3- or siRNA-ASC-transfected cells, and ASC oligomer levels were also lower in lysates from the gene-silenced strain group than in those from the wild-type group. These findings confirm that ASC oligomerization depends on NLRP3 activation and that temperature-regulated genes participate in this process.

Importantly, the temperature-regulated genes identified in this study appear to contribute to, but not solely to, the observed inflammasome-related responses. Silencing of this gene resulted in reduced cytokine production and decreased expression of inflammasome components, suggesting a modulatory role in host–pathogen interactions.

In summary, when *T. schoenleinii* shifts from room temperature (27 °C) to host-relevant temperatures (37 °C or 42 °C), heat stress upregulates the expression of temperature-regulatory factors such as TRINITY_DN3793_c0_g1 and heat shock protein genes. These changes increase fungal stress adaptation and potential virulence. Upon infection of THP-1 cells, heat-stressed fungus promotes host ROS production, cathepsin release, and K^+^ efflux, leading to the activation of NLRP3 inflammasome activity, including the upregulation of NLRP3 and ASC expression, the activation of caspase-1 activity, and the maturation and secretion of IL-1β/IL-18. Compared with the wild-type strain, the temperature-regulatory factor-silenced strain induced significantly weaker effects, indicating that this factor contributes to, but is not the sole determinant of, NLRP3 inflammasome activation. While these findings provide a molecular basis for understanding host recognition and clearance of *T. schoenleinii* and offer potential clues for targeted intervention in regionally prevalent, highly inflammatory tinea capitis, further investigations using more direct mechanistic approaches and *in vivo* models are needed to establish causal connections and confirm its relevance *in vivo*.

This study systematically reveals the molecular mechanisms of thermal stress adaptation in *Trichophyton schoenleinii* and the immunological pathways through which it regulates NLRP3 inflammasome activation. High thermal stress temperatures cause severe damage to mycelial morphology, manifested as bending, atrophy, uneven thickness, and surface roughness, indicating that thermal stress significantly affects the growth and activity of *T. schoenleinii*. Transcriptomic analysis further revealed that thermal stress induces differential expression of genes involved in ribosome biogenesis, protein processing in the endoplasmic reticulum, and heat shock proteins (TRINITY_DN3793_c0_g1), which were identified as key temperature-regulatory factors. Functional experiments confirmed that *T. schoenleinii* infection effectively activated the NLRP3 inflammasome in THP-1 cells through ROS, cathepsin B, and potassium efflux signals, promoting the maturation and secretion of IL-1β and IL-18 as well as ASC oligomerization. Temperature-regulatory factors participate in this inflammatory activation process, but their absence does not completely block inflammasome activation, suggesting the existence of multiple synergistic regulatory mechanisms. These findings elucidate the molecular link between temperature adaptation and the pathogenicity of *T. schoenleinii*, provide new insights into the unique pathogenesis of tinea capitis in Xinjiang, and lay a theoretical foundation for the development of therapeutic strategies targeting temperature regulatory factors or the NLRP3 inflammasome.

## Data Availability

The raw data supporting the conclusions of this article will be made available by the authors, without undue reservation.

## References

[B1] Albright J. M. Holian A. (2025). Contribution of particle-induced lysosome membrane permeabilization to NLRP3 inflammasome activation and mitochondrial ROS production. Toxicol. Sci. 208, 357–368. doi: 10.1093/toxsci/kfaf140. PMID: 41072925 PMC12646592

[B2] Celestrino G. A. Verrinder Veasey J. Benard G. Sousa M. G. T. (2021). Host immune responses in dermatophytes infection. Mycoses 64, 477–483. doi: 10.1111/myc.13246. PMID: 33480106

[B3] Chaudhary A. Mishra B. (2024). Systemic effects of heat stress on poultry performances, transcriptomics, epigenetics and metabolomics, along with potential mitigation strategies. World's Poultry Sci. J. 80, 1017–1053. doi: 10.1080/00439339.2024.2364884. PMID: 37339054

[B4] Chen H. Xie S. Zhou Y. Chen L. Xu J. Cai J. (2024). MEK1/2 promote ROS production and deubiquitinate NLRP3 independent of ERK1/2 during NLRP3 inflammasome activation. Biochem. Pharmacol. 230, 116572. doi: 10.1016/j.bcp.2024.116572. PMID: 39396647

[B5] Chen Y. Ye X. Escames G. Lei W. Zhang X. Li M. (2023). The NLRP3 inflammasome: contributions to inflammation-related diseases. Cell. Mol. Biol. Lett. 28, 51. doi: 10.1186/s11658-023-00462-9. PMID: 37370025 PMC10303833

[B6] Chumakova A. Vlasov I. Filatova E. Klass A. Lysenko A. Salagaev G. (2025). Application of RNA-seq for single nucleotide variation identification in a cohort of patients with hypertrophic cardiomyopathy. Sci. Rep. 15, 18788. doi: 10.1038/s41598-025-03226-x. PMID: 40442228 PMC12122699

[B7] Deng R. Chen X. Zheng D. Xiao Y. Dong B. Cao C. (2024). Epidemiologic features and therapeutic strategies of kerion: a nationwide multicenter study. Mycoses 67, e13751. doi: 10.1111/myc.13751. PMID: 38825584

[B8] Diba K. Jafari K. Alizadeh K. Aslani N. (2024). Northwest Iranian dermatophyte isolates: anthropophilic and geophilic. Curr. Med. Mycol. 10, 345184–341535. doi: 10.22034/CMM.2024.345232.1535 PMC11688585 39744343

[B9] Fabri J. H. T. M. Rocha M. C. Fernandes C. M. Persinoti G. F. Ries L. N. A. da Cunha A. F. (2021). The heat shock transcription factor HsfA is essential for thermotolerance and regulates cell wall integrity in Aspergillus fumigatus. Front. Microbiol. 12, 656548. doi: 10.3389/fmicb.2021.656548. PMID: 33897671 PMC8062887

[B10] Fillipe P. O. D. Marcelino S. G. Shellygton S. D. L. Anna D. C. P. Igara L. O. (2021). The prevalence of dermatophytoses in Brazil: a systematic review. J. Med. Microbiol. 70, 1321. doi: 10.1099/jmm.0.001321. PMID: 33533707

[B11] Hu G. Horianopoulos L. Sánchez-León E. Caza M. Jung W. Kronstad J. W. (2021). The monothiol glutaredoxin Grx4 influences thermotolerance, cell wall integrity, and Mpk1 signaling in Cryptococcus neoformans. G3 (Bethesda) 11, jkab322. doi: 10.1093/g3journal/jkab322. PMID: 34542604 PMC8527476

[B12] Leng S. Xu W. Wu L. Liu L. Du J. Yang F. (2023). NLRP3 disturbs Treg/Th17 cell balance to aggravate apical periodontitis. J. Dent. Res. 102, 656–666. doi: 10.1177/00220345231151692. PMID: 36883625

[B13] Li Q. Li J. Zhi H. Lv W. Sang B. Zhong Y. (2024). Epidemiological survey of 32,786 culture-positive dermatophytosis cases in Hangzhou from 2018 to 2023. Mycopathologia 189, 98. doi: 10.1007/s11046-024-00899-2. PMID: 39537868 PMC11561025

[B14] Liang G. Song G. She X. Shi D. Chen M. Fang W. (2026). Chinese experts consensus on the management of uncommon dermatophytosis. iFungi. doi: 10.26599/iFungi.2026.9670004

[B15] Liang G. Zheng X. Song G. Zhang M. Liu J. Zang X. (2020). Adult tinea capitis in China: a retrospective analysis from 2000 to 2019. Mycoses 63, 876–888. doi: 10.1111/myc.13102. PMID: 32395886

[B16] Miao B. Yao J. Zhang S. Guo X. Geng Y. Gong Y. (2026). Analysis of public health risks associated with pathogenic fungi in China. iFungi. doi: 10.26599/iFungi.2026.9670003 38129156

[B17] Shenoy M. M. Rengasamy M. Dogra S. Kaur T. Asokan N. Sarveswari K. N. (2021). A multicentric clinical and epidemiological study of chronic and recurrent dermatophytosis in India. Mycoses 65, 13–23. doi: 10.1111/myc.13360. PMID: 34378240

[B18] Owona A. B. Mary A. Messi N. A. Ravichandran K. A. Mbing J. N. Pegnyemb E. (2024). Biflavonoid methylchamaejasmin and Khaya grandifoliola extract inhibit NLRP3 inflammasome in THP-1 cell model of neuroinflammation. Mol. Neurobiol. 62, 1–15. doi: 10.1007/s12035-024-04365-4. PMID: 39012444

[B19] Peng N. Zhang J. Hu R. Liu S. Liu F. Fan Y. (2024). Hidden pathogen risk in mature compost: low optimal growth temperature confers pathogen survival and activity during manure composting. J. Hazard. Mater. 480, 136230. doi: 10.1016/j.jhazmat.2024.136230. PMID: 39442307

[B20] Seetan K. Khamees A. Yassin R. Y. Ba-Shammakh S. A. Moh'd A. M. (2025). Nutritional deficiencies as risk factors for incidence and treatment response of tinea capitis in children: a prospective clinical cohort study. Postgrad. Med. 137, 887–896. doi: 10.1080/00325481.2025.2609382. PMID: 41467424

[B21] Stempinski P. R. Zielinski J. M. Dbouk N. H. Huey E. S. McCormack E. C. Rubin A. M. (2021). Genetic contribution to high temperature tolerance in Cryptococcus neoformans. Genetics 217, 1–15. doi: 10.1093/genetics/iyaa009. PMID: 33683363 PMC8045695

[B22] Takashi M. Shigeo Y. Masaki H. Taketoshi F. Kazushi A. (2022). Change in dominant genotype of Microsporum canis, a causative fungus of zoonotic dermatophytosis, in Japan over the past 40 years. J. Dermatol. 49, 682–690. doi: 10.1111/1346-8138.16386. PMID: 35411631

[B23] Tettey A. P. Fujii S. Saito H. Sambongi Y. Kawai K. (2024). Thermal tolerance of larvae of seven Chironomus species and upregulation of heat shock protein-coding genes in Chironomus sulfurosus. Limnology 26, 1–9. doi: 10.1007/s10201-024-00765-6 39840382

[B24] Wu T. Sheng Y. Tian Y. Wang C. (2023). Vitexin regulates heat shock protein expression by modulating ROS levels thereby protecting against heat-stress-induced apoptosis. Molecules 28, 1–17. doi: 10.3390/molecules28227639. PMID: 38005362 PMC10675196

[B25] Xu R. Yuan L. S. Gan Y. Q. Lu N. Li Y. P. Zhou Z. Y. (2024). Potassium ion efflux induces exaggerated mitochondrial damage and non-pyroptotic necrosis when energy metabolism is blocked. Free Radic. Biol. Med. 212, 117–132. doi: 10.1016/j.freeradbiomed.2023.12.029. PMID: 38151213

[B26] Yingying S. Hongying C. Wenxiu G. Lara S. Suhong L. Lili L. (2024). Endophytic fungi improved wheat resistance to Rhopalosiphum padi by decreasing its feeding efficiency and population fitness. Ecotoxicol. Environ. Saf. 270, 115865. doi: 10.1016/j.ecoenv.2023.115865. PMID: 38134640

[B27] Zheng D. Liang T. Wu W. Al-Odaini N. Pan K. Huang L. (2023). The epidemiology of tinea capitis in Guangxi Province, China. Mycopathologia 188, 489–496. doi: 10.1007/s11046-023-00762-w. PMID: 37356056

[B28] Zhu P. Shao J. Wang R. Xiao Y. Zhou Y. Li Q. (2025). Development and clinical detection of rapid molecular diagnostic system for pathogenic dermatophytes of tinea capitis of multiple centres in China. Mycoses 68, e70008. doi: 10.1111/myc.70008. PMID: 39727089

[B29] Z. M J. Raghav T. Aaron B. Jeffrey S. F. Bibee P. K. (2024). Sociodemographic trends and financial burden of emergency department visits due to dermatophytosis in the United States. Arch. Dermatol. Res. 316, 267. doi: 10.1007/s00403-024-02980-8. PMID: 38795122

