# Supplementary material for: Study of the temperature-related factors that activate the NLRP3 inflammasome in Trichophyton schoenleinii and their activation mechanisms
Source: Front Cell Infect Microbiol. 2026 May 20;16:1823190. doi: 10.3389/fcimb.2026.1823190 (PMC13230011; doi:10.3389/fcimb.2026.1823190)
Supplement: Supplementary file 1 [file DataSheet1.pdf]

## Supplementary files

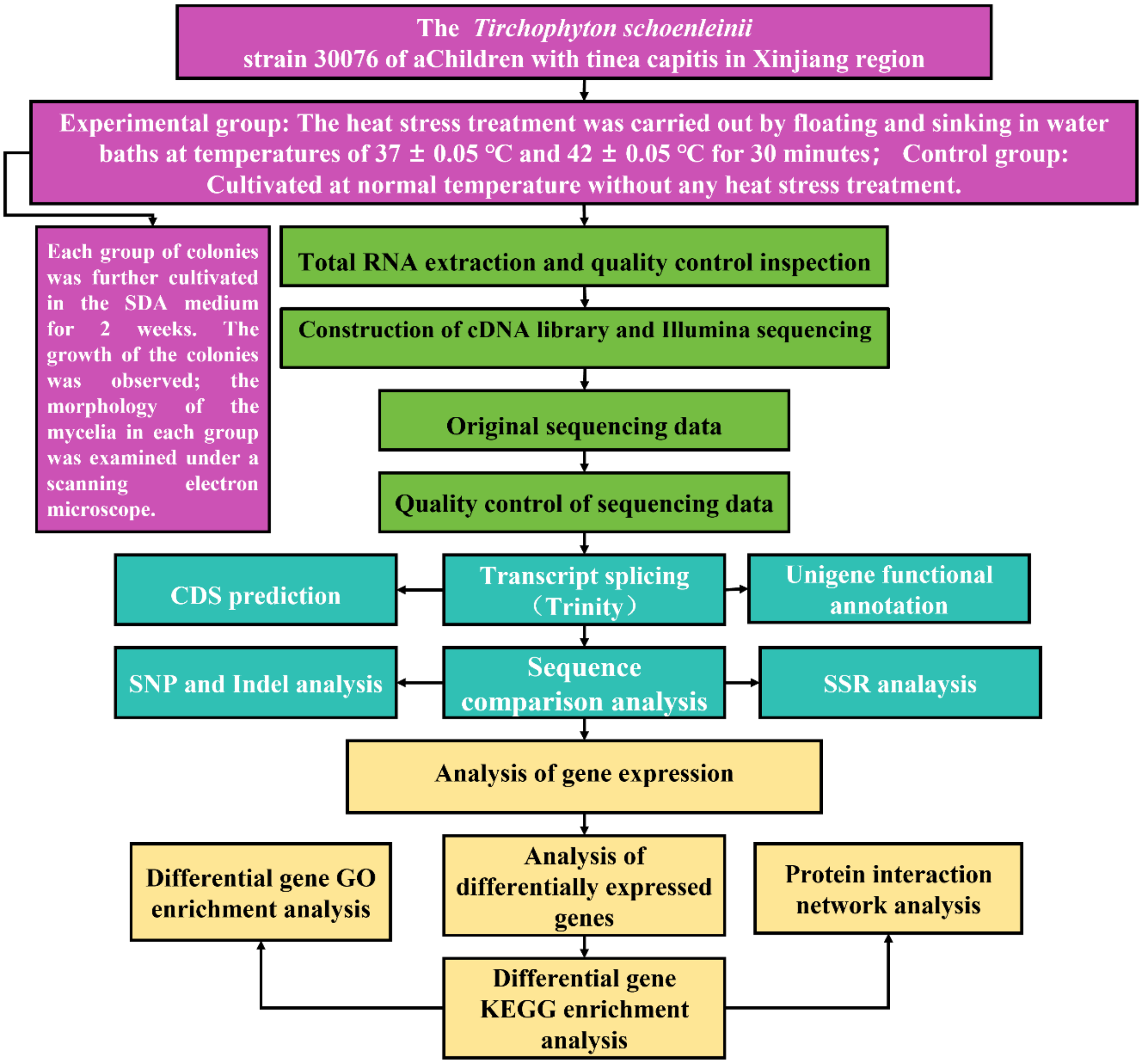


**Supplementary figure 1.** Flow chart of the experiment. Procedures used for sample preparation, RNA extraction, and sequencing. Including a schematic diagram summarizing the experimental workflow would greatly enhance the clarity of the study design.

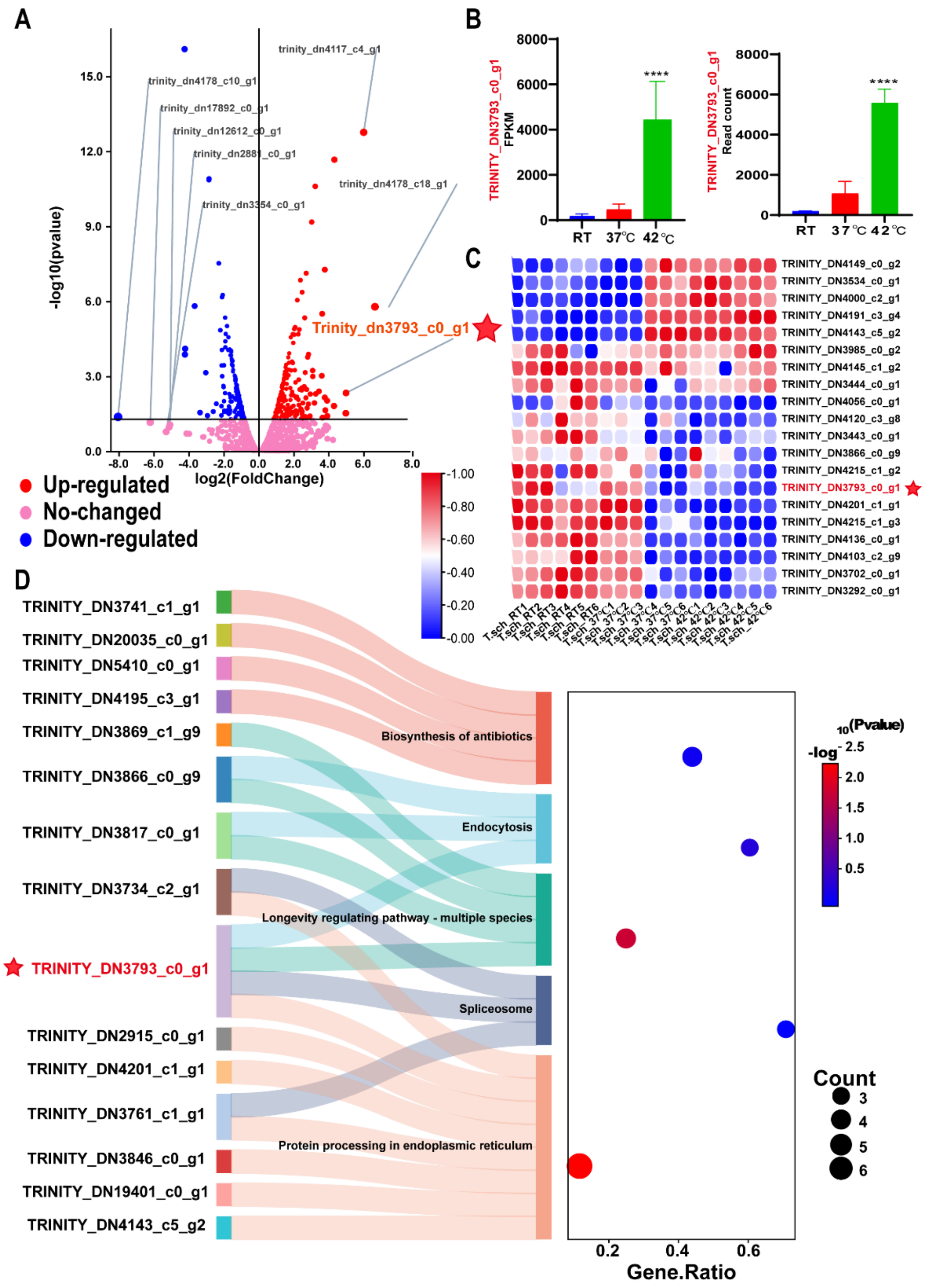


**Supplementary figure 2.** Molecular Mechanisms of *T. schoenleinii* in Response to Heat Stress: Functional Enrichment Analysis of Differentially Expressed Genes

based on transcriptome sequencing and the screening of differentially expressed genes among different temperature treatment groups, a volcano plot was used for visualization.

In the plot, the horizontal axis represents the $\log_2$ fold change in gene expression, and the vertical axis represents the negative logarithm of the adjusted P-value (-log10 padj). Upregulated genes are marked in red, downregulated genes in blue, and genes with no significant differential expression are shown as blue dots. It is clearly visible that, in the 42°C heat stress group compared with the normal temperature group, among the top eight significantly differentially expressed genes, TRINITY_DN3793_c0_g1 is one of the top three upregulated differentially expressed genes (**Supplementary figure 2**A). Second, the bar chart of gene expression level analysis shows that the expression level of this gene differs significantly under different temperature conditions, and the difference is statistically significant ($P < 0.05$). As the temperature of heat stress increases, the expression level of this gene shows a marked increase (**Supplementary figure 2B**). Third, the clustering analysis of differentially expressed genes, presented as a heatmap, shows the expression levels of this gene under different experimental conditions. Genes within clustered regions of similar colors have similar expression patterns. The expression pattern of this gene differs among groups, while within-group differences are relatively small (**Supplementary figure 2C**). Fourth, the results of biological pathway annotation and homology analysis reveal that some significantly differentially expressed genes exhibit repeated enrichment in various metabolic pathways. For example, the TRINITY_DN3793_c0_g1 gene is significantly enriched in the longevity regulatory pathway, protein processing in the endoplasmic reticulum, endocytosis, ribosomes, and other important metabolic pathways and gene functional categories. Furthermore, it participates in the expression of major heat shock proteins within these pathways.
